# Efficacy of a Vaccine Formula against Tuberculosis in Cattle

**DOI:** 10.1371/journal.pone.0076418

**Published:** 2013-10-18

**Authors:** Germinal J. Canto Alarcon, Yezenia Rubio Venegas, Luis Bojorquez Narvaez, Oscar E. Pizano Martínez, Leticia García Casanova, Susana Sosa Gallegos, Alejandro Nava Vargas, Andrea M. Olvera Ramírez, Feliciano Milian Suazo

**Affiliations:** 1 Facultad de Ciencias Naturales, Universidad Autónoma de Querétaro, Querétaro, Qro., México; 2 Productora Nacional de Biológicos Veterinarios (PRONABIVE), Distrito Federal, México; 3 Centro Nacional de Investigación Disciplinaria en Fisiología y Mejoramiento Animal, INIFAP, Ajuchitlán, Qro., México; University of Melbourne, Australia

## Abstract

“Test-and-slaughter” has been successful in industrialized countries to control and eradicate tuberculosis from cattle; however, this strategy is too expensive for developing nations, where the prevalence is especially high. Vaccination with the Calmette-Guérin (BCG) strain has been shown to protect against the development of lesions in vaccinated animals: mouse, cattle and wildlife species. In this study, the immune response and the pathology of vaccinated (BCG-prime and BCG prime-CFP-boosted) and unvaccinated (controls) calves were evaluated under experimental settings. A 10^6^ CFU dose of the BCG strain was inoculated subcutaneously on the neck to two groups of ten animas each. Thirty days after vaccination, one of the vaccinated groups was boosted with an *M. bovis* culture filtrate protein (CFP). Three months after vaccination, the three groups of animals were challenged with 5×10^5^ CFU via intranasal by aerosol with a field strain of *M. bovis*. The immune response was monitored throughout the study. Protection was assessed based on immune response (IFN-g release) prechallenge, presence of visible lesions in lymph nodes and lungs at slaughter, and presence of bacilli in lymph nodes and lung samples in histological analysis. Vaccinated cattle, either with the BCG alone or with BCG and boosted with CFP showed higher IFN-g response, fewer lesions, and fewer bacilli per lesion than unvaccinated controls after challenge. Animals with low levels of IFN-g postvaccine-prechallenge showed more lesions than animals with high levels. Results from this study support the argument that vaccination could be incorporated into control programs to reduce the incidence of TB in cattle in countries with high prevalence.

## Introduction

Bovine tuberculosis (bTB) is an infectious disease of cattle and other animal species, including man [1]. The importance of bTB is that it represents a risk to public health, causes serious economic losses to the livestock industry worldwide due to animal disposal, carcass confiscation, premature culling, low production, poor reproductive performance, and because it represents a constraint for international trade of animals [2], [3].

Mexico, as many countries around the world, counts with a national program to control and eradicate tuberculosis from cattle. This program is based on the strategy of “test-and-slaughther” where animals reacting to the tuberculin tests are eliminated. Even though this strategy has been successful in reducing the incidence of the disease in some industrialized countries [4], in developing countries, where the prevalence of the disease is especially high, the success has been limited. In Mexico, as in most of Latin America, tuberculosis concentrates in populations of dairy cattle, where “test-and-slaughter” is unfeasible because elimination of animals represents less milk for the human population and there is a cost for the producer due to lack of compensation for disposal of animals and the cost of importing replacements from abroad. Therefore, the use of a vaccine to control and, in the long run, eradicate bTB could be an important addition to control programs already established [5], [6].

Studies evaluating the efficacy of vaccines for tuberculosis in cattle have focused on the use of the bacillus Calmette–Guerin (BCG) strain, an attenuated strain of *M. bovis* used in humans since 1921 [7]. BCG is the only licensed vaccine against human TB; therefore, it is likely that any vaccine or vaccination strategy in cattle is based on this strain [8]. As a matter of fact, numerous studies have already shown that BCG induces a significant level of protection against development of lesions in animals vaccinated and challenged with virulent strains [9], [10], [11], [12], [13]. Subcutaneous and oral administrations of BCG, the use of BCG in a lipid matrix, and different BCG strains have shown protection [14], [15]. It has been shown that: low doses of BCG [10^3^–10^6^ colony forming units (CFU)] induce better protection than higher doses [5], pre-exposure to environmental *Mycobacterium* can negatively affect vaccine efficacy [16], vaccination of neonatal calves induced a higher level of immunity than that observed in calves vaccinated at 5–6 months of age [11], [12], [17], and that boosting with culture filtrate proteins (CFP) of *M. bovis*, plus adjuvant induce a better Th1 response. Conclusions of these studies are that vaccine protection occur by reducing the number of animals with lesions, the number of lesions per animal and bacteria load per lesion [11], [12].

Vaccination against tuberculosis has been used in humans for many years; however, it has never been officially used in control programs in cattle because the immune response interferes with the tuberculin test, which in most countries is the only official test to estimate prevalence. Nevertheless, on the one hand, it has been shown that only a few vaccinated animals react to the comparative cervical test, since BCG induces response to both avian and bovine-PPD [11], [12], and, on the other hand, this limitation is irrelevant in countries with no eradication programs or countries with large populations of dairy cattle where the tuberculin test is rarely used. More than that, nowadays there are at least two antigens, ESAT6 and CFP10 that can differentiate between TB infected and TB vaccinated animals [18]. In Mexico, the bTB Control and Eradication Program has been successful in beef cattle, reducing the prevalence to less than 0.5% in about 85% of the national territory; however, in dairy cattle the program has failed since only about 30% of the farms have some partial participation in the program [19]; therefore, tuberculin testing is not performed in a routine bases. Nevertheless, dairy farmers around the country are willing to participate more in the bTB National Program as far as herds are not labeled as quarantined and animals reacting to the tuberculin tests are not removed. As a matter of fact, a recent survey in dairy regions showed that more than 90% of the farmers would be willing to participate in the program if a vaccine is used. Therefore, the aim of this study was to evaluate the efficacy of a BCG vaccine formula against bovine tuberculosis under experimental settings to provide to the National Program with a new tool against this problematic disease.

## Materials and Methods

### Experimental Animals

Thirty, five to six months old, 150 kg weight calves free of TB coming from a TB-free area were included in the study. Animals were divided into three groups and allocated in isolation in high-security confinement units, fed a diet based on grain and forage with free access to water during the experimental period. All procedures received approval of the institutional committee for the protection of animals used in research (*Comité del Instituto Nacional de Investigaciones Forestales, Agrícolas y Pecuarias para el Cuidado de los Animales usados en Investigación*).

### Vaccine Strain

The *M. bovis* BCG Phipps strain was used. This strain was selected because it induced the best protection against challenge in a mouse model where 10 BCG substrains were compared [20]. Bacteria were grown in Dorset-Henley medium supplemented with 0.25% (wt/vol) glucose. Cultures were incubated at 37°C for four weeks. Bacteria were then harvested and shacked continuously at 200 rpm/2 h. Bacterial count was determined by serial dilution on Stonebrink media tubes supplemented with sodium piruvate. CFU were determined after 21 days of incubation. Vaccine was then adjusted to contain 10^6^ bacteria per ml and stored at −70°C until use.

### Culture Filtrate Proteins (CFP)

*M. bovis* CFP were prepared as follows. *M. bovis* AN5, the strain used to produce the PPD for skin testing cattle in México was grown as a pellicle in Dorset Henley medium for 8–10 weeks. Cultures supernatant was sterile-filtered twice through a 0.22 µm filter and tangentially filtered using a 10 kDa molecular weight cut-off filter (Pelicon Membrane, Millipore, Inc., USA). The CFP was stored at 4°C until use. Protein content of the culture filtrate was determined using a Biuret Reagent, and protein adjusted to 200 µg/ml (P2B010A05, membrane type: PBGC). CFP was prepared by mixing *M. bovis* CFP with Polygen TM (MVPLaboratories, Inc.). The adjuvant was then added to a final concentration of 10% (v/v).

### Study Design and Vaccination

Animals were allocated throughout a completely randomized experimental design into three groups: the control, unvaccinated group, the BCG vaccinated and the BCG vaccinated and CFP boosted group. Vaccinated groups were inoculated subcutaneously on the neck with a dose of 10^6^ colony forming units (CFU) of the BCG strain in 2 ml diluent. The boosted group was vaccinated with the BCG strain and four weeks later with a *M. bovis* CFP, 400 µg/ml, plus polygen.

### *M. bovis* Challenge

The challenge inoculums consisted of a mid-log-phase of *M. bovis* grown in Stonebrink media supplemented with piruvate. The challenge strain was obtained from the lymph node of a dairy cow in 2010. To harvest the bacilli, bacteria were pelleted by centrifugation at 750 *g* and washed twice with phosphate-buffered saline solution (PBS, 0.01 M, pH 7.2). Bacilli were then perfectly homogenized in PBS and shacked by glass beads continuously at 200 rpm for 1 h. The homogenate was sterile-filtered twice throughout a 40 Watman filter and diluted to the established doses (5×10^5^ CFU) for use as inoculum in 2 ml of PBS. Bacilli counting were performed by serial dilution in Stonebrink media plates. Calves were challenged by aerosol as described by Palmer *et al.,* [21]. Briefly, previous to challenge, animals were inoculated 0.25 mg/kg of Xylazine hydrochloride 10% IM, and then restrained in a head chute. The challenge inoculum was delivered by nebulization into a mask covering the nostrils and mouth. The nebulization apparatus consisted of a compressed air tank and a commercially available aerosol delivery system (Equine Aeromask, Trudell Medical, London, Ontario, Canada) comprised of a jet nebulizer (Whisper Jet, Marquest Medical Products, Englewood, CO, USA), holding chamber and mask. Compressed air (25 psi) was used to jet nebulize inoculum directly into the holding chamber. Upon inspiration, the nebulized inoculum was inhaled and delivered through a one-way valve into the mask and directly into the nostrils during 8 min. The inoculums chamber was washed with 1 ml of PBS every time previous to the next delivery. Experimental infection was done in an isolated area to prevent contamination. Personnel used appropriate protective equipment, including full-face respirators with HEPA-filtered canisters and disposable protective clothing to prevent exposure to aerosolized *M. bovis*. The workplace and instruments were decontaminated with a 10% phenol solution and recipients used sterilized in autoclave.

### Blood Collection and IFN-g Release Assay

Blood samples were collected at day 0, 15, 40 and then about every two weeks until the end of the experiment, eight months after vaccination. These samples were shipped to the laboratory at ambient temperature and processed within 2 h of collection. To measure T cell response in whole-blood cultures after 16-h *in vitro* antigen stimulation, a commercial bovine IFN-g microplate enzyme-linked immunosorbent assay (ELISA; Bovigam; Commonwealth Serum Laboratories, Australia) kit was used. The kit was used according to the manufacturer instructions with a few modifications in reagent concentration. Briefly, samples were collected from the coccygeal vein and placed into heparin tubes. From there, 750 µl of whole blood were incubated in microplates, in duplicate with 50 µl for each antigen (bovine-PPD, avian-PPD). Negative control wells with phosphate-buffered saline (PBS) were included for each animal tested. Positive controls containing 50 µl of pokeweed mitogen of a 1 µg/ml concentration solution (Sigma–Aldrich, United Kingdom) were also included before incubating in a humidified 5% CO_2_ incubator at 37°C for 20 h. Optical densities (OD) of PBS from the control wells were used to normalize individual readouts and to calculate optical density. Final OD readings were obtained by subtracting sample readings from PBS control readings.

### Slaughter and Lesion Counting

Six months after challenge, experimental animals were sent to slaughter for carcass inspection, visible lesions counting and tissue sampling for histopathological and microbiological analysis. In order to perform a careful inspection of carcasses, animals were slaughtered in a three-week period; slaughtering three animals per day. Although all organs were reviewed, especial attention was place on lymph nodes: retropharyngeal, mediastinal and tracheobronquial, and lungs, liver and spleen. Lymph nodes were cut in half and all lesions counted. Liver, spleen and lungs were sliced in pieces of about 1 cm wide to count all visible lesions. Special care was focus in no counting the same lesion twice in both sides of the slice. Animals were slaughtered according to recommendations in the *NORMA Oficial Mexicana NOM-033-ZOO-1995, Sacrificio humanitario de los animales domésticos y silvestres* (Official norm for the humanitarian slaughter of domestic and wildlife animals), which is the legal document ruling this practice. Briefly, previous to slaughter, animals were kept in a quite environment, managed with care throughout the chute on the way to the final step and finally shut in the forehead with an air pistol.

### Statistical Analysis

The average IFN-g concentration (optical densities raw data) per group for every sampling period was compared with a one-way ANOVA test. The number of lesions per animal was first categorized into 5 groups according to the number of lesions in each experimental animal (1 = 0, 2 = 1 to 30, 3 = 31 to 100, 4 = 101 to 1000 and 5>1000) and the groups compared with the H statistic in the Kruskal-Wallis test. The association between the different experimental groups and the proportion of TB-compatible lesions was estimated throughout a chi-square test. Comparison of the number of bacilli between vaccinated and control groups were performed throughout the nonparametric Kruskal-Wallis test. All analyses were performed in the SPSS software.

## Results

### IFN-g Release

**Figure 1:**
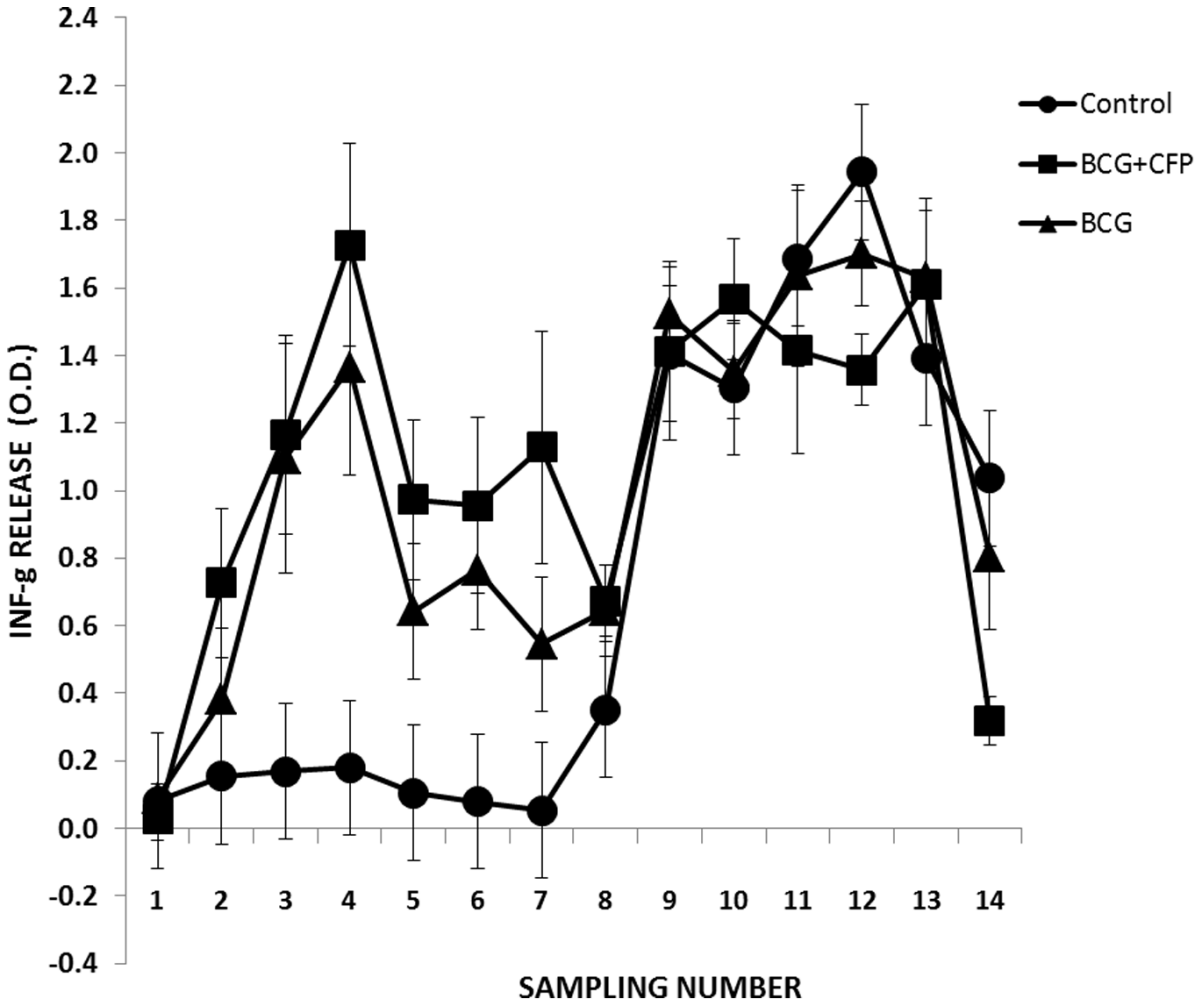
IFN-g response after vaccination and challenge. Plasma from whole-blood cultures of vaccinated (BCG and BCG+CFP) and unvaccinated (control) animals were stimulated *in vitro* with bovine PPD and avian PPD and assayed for IFN-g. Challenge was delivered in sampling number 8. Sampling was performed at days 0, 15, 40 and then about every two weeks until the end of the experiment.

IFN-g in plasma is used as a surrogate of vaccine efficacy against tuberculosis in cattle [22], [9], [10], [5], [22], [23], [13], [8], [24]. In this experiment, IFN-g response to bovine-PPD was measured at different time-periods after vaccination and after challenge (Figure 1). No significant difference was observed between groups at day 0 (P = 0.70). At day 30, even though the difference between the vaccinated and the control groups was not statistically significant (P = 0.13), the concentration of IFN-g was higher in the vaccinated than in the control group. From the second period on, the difference between the vaccinated and the control groups was statistically significant (P<0.05) until day 90, the challenge date. After challenge, no difference between groups was observed (P>0.05) until day 230, just previous to slaughter, where the control group had a significant higher concentration than the vaccinated groups (P = 0.03).

### Lesion Counts

**Figure 2:**
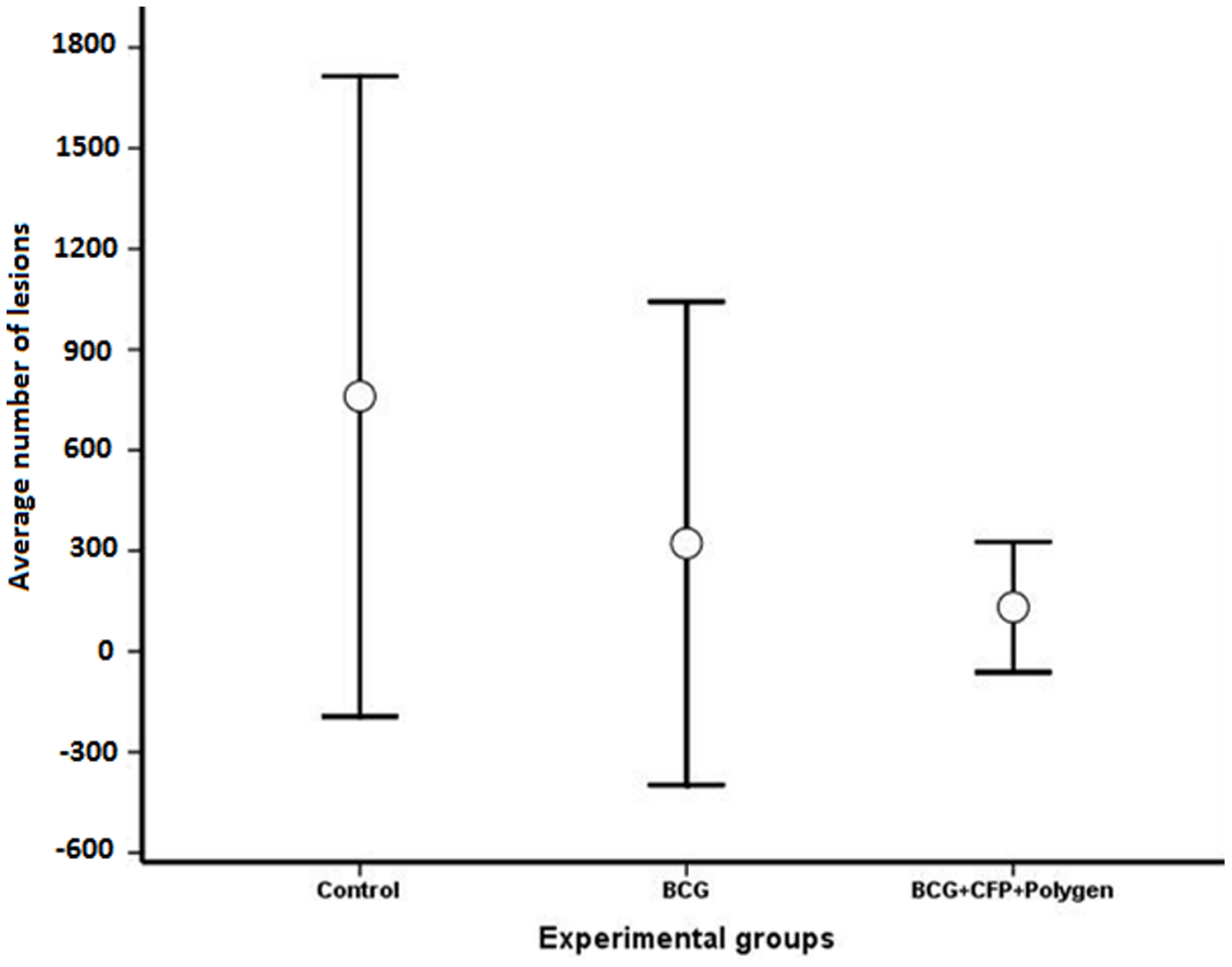
Total number of lesions per group in calves vaccinated (BCG and BCG+CFP) and unvaccinated (control) against tuberculosis. This number includes lesions from lymph nodes (mediastinal and trachebronchial) and lungs.

All animals were sent to slaughter 6 months after challenge for carcass inspection and visible lesions counting; except for one animal in the BCG+CFP group and two animals in the BCG group, all animals showed at least two visible lesions in at least one organ. The average number of lesions per group (lesions from all organs) is shown in Figure 2. Since normality was difficult to accomplish given the data values, which ranked from 0 to more than 1000, a non-parametric rank test (Kruskal-Wallis) was used. No difference between the vaccinated and the control groups was observed (Chi-Square test = 2.402, 2 df and P = 0.301, with a mean rank of 18.75, 13.0 and 14.7 for the control, the BCG and the BCG+CFP+Polygen groups respectively); however, the average number of lesions was much lower in the vaccinated groups (n = 322±720 and 132±726, for the BCG and the BCG+CFP groups, respectively) than in the control group (n = 760±954.6). It is important to mention that in the BCG group, a single animal with 2,300 lesions increased considerable the average of lesions for that group. In the control group, four animals had more than 1,000 lesions. Similar results were observed when counting lesions per organ; the higher number of lesions was observed in the mediastinal and the tracheobronchial lymph nodes (Table 1).

**Table 1:** Average number of visible lesions per affected organ in vaccinated (BCG and BCG+CFP) and unvaccinated (control) calves challenged with a field strain of *M. bovis*.

Organ	Group	Average number of lesions	Standar deviation	IC_95%_	Min.	Max.
**Mediastinal lymph node**	Control	316^a^	432	7;625	0	1000
	BCG	147^a^	406	−144;437	0	1300
	BCG+CFP	48^a^	76	−6;102	0	250
**Tracheobronchial** **lymph node**	Control	351^a^	473	12;689	0	1000
	BCG	114^a^	218	−41;270	0	550
	BCG+CFP	20^b^	31	−2;42	0	100
**Lungs**	Control	91^a^	130	−1;184	2	437
	BCG	64^a^	155	−50;172	0	500
	BCG+CFP	61^a^	154	−46;174	0	500

### Histopathology

Four samples from each experimental animal were sent for histopathological analysis. The average number of bacilli in 100 fields with a 40× lens per group is shown in Table 2. Comparison of groups was performed throughout the ranks Kruskal-Wallis test. No significant difference between groups was observed (Chi-Square = 3.164, 2 df, P = 0.206); however, the average number of bacilli observed in the control group (4.8) was considerable higher than that observed in the vaccinated groups (0.8 for the BCG and 2.0 for the BCG+CFP groups). In relation to the number of samples with TB-compatible lesions (Table 3), the proportion was higher in the control (55%) than in the vaccinated groups (45% for the BCG and 36% for the BCG+CFP groups); however, the association in a chi-square test was not significant (P = 0.27).

**Table 2:** Average number of *M. bovis* bacilli per 100 fields, 40× lens in vaccinated (BCG and BCG+CFP) and unvaccinated (control) calves challenged with a field strain of *M. bovis*.

Group	Average number of bacilli per 100 fields, 40× lens	Estándar deviation	IC_95%_	Min.	Max.
**Control**	15a	30.04	−6.5;36.5	0	75
**BCG**	6a	12.85	−3.2;15.2	0	6
**BCG+CFP**	2.8a	3.2	0.52;5.1	0	20

**Table 3:** Number and proportion of positive tissue-samples to the presence of lesions TB-compatible detected in histopathological analysis in vaccinated (BCG and BCG+CFP) and unvaccinated (control) calves against tuberculosis and challenged with a field strain of *M. bovis*. At least one sample from each affected organ was included in the analysis.

Group	Positive	Proportion of positive	Total
**Control**	22	55	40
**BCG**	18	45	40
**BCG+CFP**	14	37	38

### Tuberculin Conversion

All experimental animals were tuberculin tested at day 0, 8 weeks post vaccination and 20 weeks post challenge (one week previous to slaughter). All animals were tuberculin negative in the first test. In the second test, 1, 9 and 9 animals from the control, BCG and BCG+CFP groups respectively, were positive. In the third test, all animals were positive.

## Discussion

Efficacy of the BCG vaccine in reducing dissemination of *M. bovis* in cattle has been variable [25], [26], [27], [9], [10], [28], [5], [23]. Different factors have been associated to this variability: doses, the inoculation route and the BCG strain used [29], age to vaccination and previous exposure to environmental *Mycobacterium* [12]. In our study we used a 10^6^ CFU doses, based on reports from previous studies [5], [9], [30].

In relation to the vaccination route, we used the SC route. Other studies have used the oral and the nasal routes; however, those are more impractical in the field [10], [23]. The most frequently used strains in experimental studies in cattle have been the Pasteur and the Danish strains [31], [32], [33], [30], there is general consent that all strains provide similar results. In this study we used the Phipps strain because in a previous study with ten substrains tested in a mouse model, it was the one that conferred the best protection against TB lesions in lung [20]. To prevent bias in the immune response from previous exposure to environmental *Mycobacterium* [34], [16], only calves negative to the tuberculin test and the IFN-g test were used.

Doses and Challenge routes play an important role in the pathogenesis of TB after vaccination [1]. In experimental conditions, different routes have been used: oral, intranasal, intratracheal and aerosol [35], [29], [36]. In our study the aerosol route was used because it better resembles natural conditions of infection; however, our doses was higher (5×10^5^ CFU) that that used by others. We were interested in a more aggressive dose, and a longer period post challenge-slaughter to have more notorious lesions and make a better contrast between vaccinated and unvaccinated animals.

Some studies have shown that boosting induces better protection against TB in cattle [37], [38], [39], [13], [40], [41], our results agree with those reports, the boosted group of animals had the highest concentration of IFN-g, the lowest number of lesions at slaughter and a the lowest number of bacilli in affected tissue.

**Figure 3:**
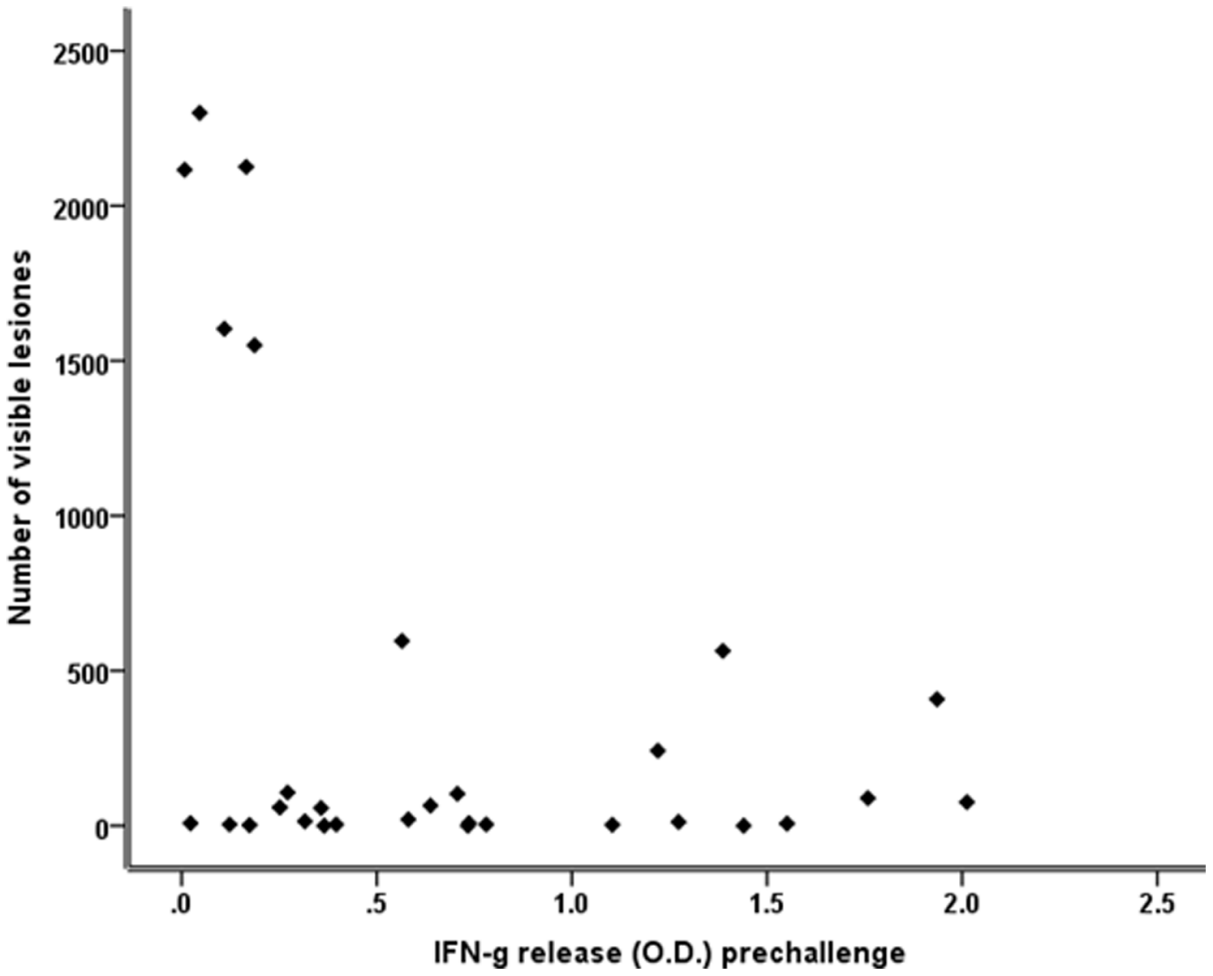
Correlation of the total number of lesions with the average IFN-g response postvaccination-prechallenge (optical densities) of calves vaccinated and unvaccinated against tuberculosis. Correlation was performed ignoring vaccination status.

IFN-g has been used as a surrogate for vaccine efficacy [9], [11], [42], [35], [43]; however, this asseveration has been questioned [44], [45], [46]. In our study, when the average of all IFN-g values postvaccination-prechallenge was correlated with the total number of lesions, it was observed that the five animals with the highest number of lesions (>1500) were from those with the lowest IFN-g concentration (<0.3 O.D.), clearly suggesting a negative correlation (Figure 3).

In experimental studies it is always difficult to come up with a single and definitive value reflecting vaccine efficacy; however, if we base efficacy in terms of the total number of lesions and consider the control group as the reference group (0% efficacy), the relative efficacy is 58% [efficacy = 1−(number of lesions in the vaccinated group/number of lesions in the control group) × 100] for the BCG group and 83% for the BCG+CFP group.

No significant differences were observed in the number of animals with visible lesions at slaughter between the vaccinated and the control groups, as reported in other studies [30], [47], [48]. This could be a consequence of the high doses of bacilli and the route of inoculation of the challenge inoculum in our study, we believed that both influenced the implantation of infection and, consequently, favor the development of lesions.

There are some reports about a low proportion of vaccinated animals responding to the tuberculin test, in our study, two months after vaccination 90% of the vaccinated animals were positive, a similar result to that reported for at least another study [49]. Therefore, there is an urgent need for tests that perform differential diagnosis if the vaccine is to be used in the field.

Even though no significant differences between vaccinated and unvaccinated groups of animals were observed in this study, our results support the hypothesis that the vaccine has the potential to reduce the dissemination of TB in cattle. We believe that no significant difference between groups was more a consequence of the high variability of lesions provided for one or two animals in the vaccinated groups, than for a real failure of the vaccine. In general, the average number of lesions in the vaccinated groups was considerable lower than that in the control group; however, the high standard deviation coming from one or two animals with a high number of lesions caused failure to detect significant difference between groups. In real life, it is highly probable that some animals are more susceptible to suffer infection and to develop disease for different reasons, some known (genetics for example) and some unknown. In our study, the high number of lesions in some vaccinated animals could have been due to factors such as natural susceptibility to infection, the high number of bacilli in the challenged doses and the method used to deliver the challenge inoculums. In natural conditions, hardly the doses of infection is going to be as high, and hardly the pressure of bacilli into the respiratory tract is going to be 25 psi. Therefore, in order to know how useful the vaccine is in control programs, field trials need to be performed.

## Conclusion

This study shows that the BCG vaccine, alone or in combination with a CFP boost, has the potential to reduce tuberculosis dissemination in cattle by reducing the number of lesions and the bacterial load per lesion. However, in order to make definitive conclusions about the usefulness of the vaccine in programs against TB in the field, it is necessary to perform long term field trials.
